# Cluster of 19 cases of eosinophilic meningitis due to *Angiostrongylus cantonensis* in Lifou, New Caledonia: an outbreak investigation

**DOI:** 10.1186/s40249-026-01498-7

**Published:** 2026-09-01

**Authors:** Yves-Marie Ducrot, Jean-Sébastien Mora, Morgane Bahu, Alexandre Bourles, Matthieu Pot, Thibaut Mathieu, Antoine Biron, Nicolas Molko, Alexandre Duvignaud, Beatrice Nickel, Julien Colot

**Affiliations:** 1 Department of Health and Community Services, Loyalty Islands Province, Lifou, New Caledonia; 2 Cardiology Department, Centre Hospitalier Universitaire de La Réunion, Saint-Pierre, France; 3 Medical and Environmental Bacteriology Group, Institut Pasteur of New Caledonia, Noumea, New Caledonia; 4 Microbiology Laboratory, Gaston-Bourret Territorial Hospital Center of New Caledonia, Noumea, New Caledonia; 5 Neurology Department, Gaston-Bourret Territorial Hospital, Nouméa, New Caledonia; 6 Department of Infectious Diseases and Tropical Medicine, Division of Tropical Medicine and Clinical International Health, CHU de Bordeaux, Bordeaux, France; 7 National Institute for Health and Medical Research (INSERM) UMR 1219, Research Institute for Sustainable Development (IRD) EMR 271, University of Bordeaux, Bordeaux Population Health Research Centre, Bordeaux, France; 8 Swiss Tropical and Public Health Institute, Allschwil, Switzerland; 9 University of Basel, Basel, Switzerland

**Keywords:** Eosinophilic meningitis, *Angiostrongylus cantonensis*, *Platydemus manokwari*, Angiostrongyliasis, Outbreak, Emerging infectious disease, New Caledonia

## Abstract

**Background:**

Eosinophilic meningitis due to *Angiostrongylus cantonensis* (AC) is an emerging parasitic disease worldwide but remains endemic in the Pacific region. In New Caledonia, sporadic cases occur each year, and the route of transmission is often unclear. In January 2023, an unusual cluster of three cases occurred in a village on Lifou Island following week-long community festivities, leading to an epidemiological investigation to determine the outbreak scope, the source of contamination, and inform public health interventions.

**Methods:**

We conducted a retrospective epidemiological and environmental investigation in one month after the suspected exposure. Cases were defined according to international criteria and classified as confirmed, probable, or suspected. Clinical data, attendance at the festivities and food exposures were collected from medical records and interviews. Environmental observations focused on potential contamination sources in food preparation areas. Serological analyses were performed in symptomatic and asymptomatic exposed individuals. Descriptive statistics were used to summarize demographic, clinical, and exposure characteristics.

**Results:**

Nineteen cases were identified, including three confirmed and sixteen suspected cases; no probable cases were identified. Headache was the predominant symptom, often associated with vomiting or paresthesias. All cases attended festivities on December 27, 2022 suggesting a common exposure during that day. The median incubation period was 3 weeks (IQR: 2–4). Two exposure hypotheses were identified: beverages diluted with rainwater and contamination of blended fruit juice. Environmental investigations revealed rats and gastropods around rainwater reservoirs and food preparation areas, including on utensils left to dry outdoors. No AC DNA was detected in water samples. Serology for AC was positive in all confirmed cases (3/3), in 13 of 14 tested suspected cases, and in all ten exposed asymptomatic individuals tested 9 months after the outbreak.

**Conclusions:**

This investigation highlights the diagnostic and epidemiological challenges of neuroangiostrongyliasis in endemic areas, where mild presentations often remain unconfirmed and where the value of serological testing is limited by the lack of baseline seroprevalence data. The findings support the hypothesis of accidental incorporation of a heavily infected gastropod into blended fruit juice during preparation, potentially facilitated by open-air kitchen practices in rural Pacific settings.

**Supplementary Information:**

The online version contains supplementary material available at https://doi.org/10.1186/s40249-026-01498-7.

## Background

*Angiostrongylus cantonensis* (AC), the rat lungworm can accidentally infect humans, leading to a clinical syndrome known as eosinophilic meningitis (EM). After an incubation period typically ranging from 6 to 35 days [1], the disease most often presents with persistent headaches, erratic pain in the spine and limbs, and occasionally motor or sensory deficits or mild meningeal signs. Cerebrospinal fluid (CSF) analysis typically reveals eosinophilic pleocytosis. These manifestations result from the migration of larvae in the central nervous system, where they eventually die. In cases of severe infection, the associated inflammatory reaction may lead to neurological sequelae, impaired consciousness, or even death [1]. Current treatment recommendations favor early administration of corticosteroids combined with albendazole for approximately 2 weeks [2].

The life cycle of *A. cantonensis* involves mollusks as intermediate hosts, which become infected by ingesting rat feces containing first-stage larvae. Within the mollusk tissues, larvae develop into infective third-stage larvae. The cycle is completed when rats consume infected mollusks. Larvae may also be transferred to other animals such as planarians, lizards, or crustaceans, which act as paratenic hosts [3].

Human infection is usually associated with the intentional or accidental consumption of raw or undercooked mollusks or paratenic hosts. However, contamination of fresh produce or beverages through contact with infected mollusks has also been suggested [4].

Eosinophilic meningitis due to *A. cantonensis* is considered an emerging disease originally described in Southeast Asia. Since the 1940 s, it has progressively spread throughout the Pacific region, likely due to the dispersal of infected rats transported on commercial and military ships [5]. The parasite is now reported in several regions worldwide, including Europe [6–8].

New Caledonia is a French overseas territory of approximately 280,000 inhabitants located between Australia and Fiji. New Caledonia was among the first Pacific territories affected during this geographic expansion [9, 10]. Currently, around ten sporadic cases are diagnosed annually, mainly among Melanesian populations living in rural areas and typically during the end of the cool and dry season [11]. The exact mode of contamination is rarely identified. In 1976, Ash suggested that terrestrial flatworms might play a role in transmission during the harvesting season of raw vegetables, possibly explaining the marked seasonality observed in New Caledonia [12].

In January 2023, three inhabitants of a small village in New Caledonia were hospitalized for neuroangiostrongyliasis. All of them had participated in several days of community festivities during the Christmas period. Other attendees also reported symptoms compatible with the infection. An epidemiological investigation of this cluster was therefore initiated to determine the outbreak scope, the source of contamination, and inform public health interventions [13].

## Methods 

### Study setting

Lifou Island has a population of about 9800 people, predominantly Melanesian, whose lifestyle still preserves important traditional elements organized around customary practices, yam cultivation, and religion. The region has a tropical maritime climate characterized by a hot and rainy season from November to April and a cooler and drier season from May to October.

The investigated village is located on Lifou Island, a part of the Loyalty Islands in New Caledonia. Lifou is the largest island of the Loyalty Islands Province and lies approximately 190 km east of the main island of New Caledonia. The village has approximately 80 inhabitants. Housing mainly consists of wooden or corrugated metal constructions, with separate buildings for kitchens, sanitary facilities, and traditional thatched huts. Most households have access to electricity and running water.

Two primary healthcare centers are located on the island; however, in case of medical or surgical emergencies, patients are transferred to the territorial hospital located in Nouméa.

### Case definition and case-finding

Case definitions were based on the criteria proposed by the International Angiostrongylus and Angiostrongyliasis Alliance [14]. (i) A confirmed case was defined as the presence of compatible symptoms together with a positive PCR for *A. cantonensis* in cerebrospinal fluid (CSF). (ii) A probable case was defined as compatible symptoms associated with eosinophilic meningitis and at least two minor criteria (compatible exposure, positive serology, or peripheral blood eosinophilia). (iii) A suspected case was defined as compatible symptoms together with at least one minor criterion.

The minor exposure criterion corresponded to participation in meals during the festivities. Because this exposure criterion alone could classify a symptomatic individual as a suspected case, we further stratified suspected cases according to the presence of additional minor criteria, namely peripheral eosinophilia (> 500/mm^3^) and/or positive serology. Suspected cases were therefore categorized as follows: (i) low suspicion: 1 of 3 criteria; (ii) moderate suspicion: 2 of 3 criteria; (iii) high suspicion: 3 of 3 criteria. This additional stratification was introduced for descriptive purposes and to better reflect the level of diagnostic probability among suspected cases.

The investigation began on February 2 approximately 1 month after the suspected exposure period and was therefore largely retrospective, relying on medical records and participant interviews. The main difficulty was that the festivities had taken place over an entire week, with meals and activities organized both morning and evening.

**Fig. 1:**
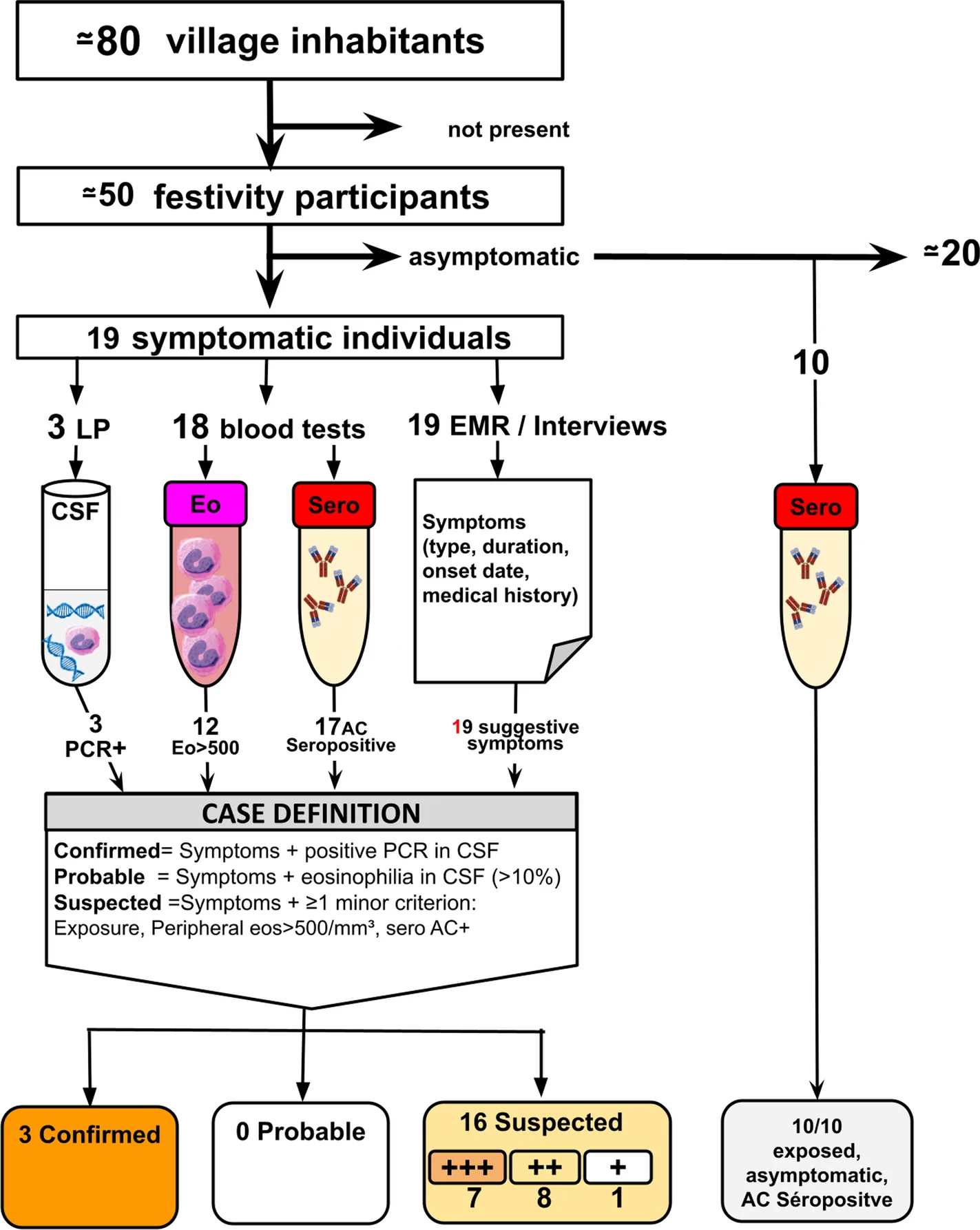
Flowchart of exposure, clinical investigation, and case classification during the angiostrongyliasis cluster in Lifou, New Caledonia. *LP* lumbar puncture, *CSF* cerebrospinal fluid, *PCR* polymerase chain reaction, *Eo* eosinophils, *Sero* serology, *EMR* Electronic Medical Record. Confirmed case: symptoms with positive PCR in CSF. Probable case: symptoms with eosinophilia in CSF (> 10%). Suspected case: symptoms with ≥ 1 minor criterion (exposure, peripheral eosinophilia > 500/mm^3^, or positive serology)

For the three hospitalized and confirmed cases, detailed hospital records were retrieved. Electronic medical records of residents who had consulted at the nearest health center between 1 and 6 weeks after the end of the festivities were systematically reviewed to identify symptoms compatible with neuroangiostrongyliasis. In addition, an investigator visited the village for several days to interview the majority of participants and identify potential cases who had not sought medical care (Fig. 1).

### Investigation of food, water, and environmental exposures

Given the clustered occurrence of cases, and particularly the nearly simultaneous onset of the three confirmed cases, the hypothesis of a single source of contamination was considered.

The investigator conducted systematic interviews with participants and organizers to reconstruct the foods and beverages served during the different meals. Photographs taken during the festivities also helped document the food items consumed.

Each food or beverage was classified according to an estimated level of risk based on its origin, method of preparation, and degree of cooking. Industrially processed products and thoroughly cooked foods were considered to present a low risk (Table S1).

A detailed timeline of the presence of the individuals meeting the case definition was reconstructed for each day of the festivities (Table S2). Food items not consumed on those specific days were subsequently excluded from the list of potential exposures. No food leftovers could be analyzed more than 2 weeks after the suspected date of contamination.

Epidemiological investigations quickly revealed that all participants had consumed rainwater collected from household rainwater harvesting systems. These reservoirs were therefore identified as potential sources of contamination. Water samples (750 ml) were collected on February 9, 2023 from the surface of the reservoirs after agitation of the bottom sediment. Samples were stored at ambient temperature and transported to the microbiology laboratory in Nouméa the following day.

Environmental assessment was conducted on site by the investigator and included the search, through witness reports and direct observations, for the presence of rats and mollusks near areas where fruits and vegetables were harvested, as well as near locations used for food preparation and storage.

Dissection and PCR analysis of mollusks could not be performed at the time of the study because of logistical constraints, particularly difficulties related to transport and sample preservation.

### Laboratory investigations

Clinical blood samples were collected from the three confirmed cases at the Territorial Hospital Center of New Caledonia (Nouméa), from 15 of the 16 suspected cases at the Lifou Medical Center in March 2026, and from 10 exposed but asymptomatic individuals in September 2026. One suspected case could not be sampled. Serum samples were available from 14 of the 16 suspected cases, as one patient had only undergone a complete blood count during the acute phase of illness. At the Lifou Medical Center, blood collected in dry tubes was centrifuged on site, and serum was separated and transported at 4 °C to the Territorial Hospital Center within 24 h together with EDTA blood samples for complete blood counts. Serum samples from both collection sites were subsequently stored at −80 °C before being shipped on dry ice to the Swiss Tropical and Public Health Institute (Swiss TPH), Basel, Switzerland.

At the Territorial Hospital Center, CSF samples from the three hospitalized patients were analyzed by PCR according to the methodology described by Melot et al. [11]. Complete blood counts were performed on all available EDTA blood samples.

At the Swiss TPH, serological testing was performed using an in-house Western blot assay with antigens derived from adult *A. cantonensis* worms and detection of the diagnostic 31-kDa antigenic band by immunoblot [15]. Potential cross-reactivity with *Toxocara canis* and *Strongyloides stercoralis* was also assessed. Serological analyses were performed on serum samples from all three confirmed cases, 14 suspected cases, and the 10 exposed but asymptomatic individuals.

Environmental water samples were examined microscopically before and after centrifugation at the Territorial Hospital Center and subsequently analyzed by PCR using the same molecular protocol as that used for the CSF samples.

### Statistical analysis

Descriptive statistics were used to summarize demographic, clinical, and exposure characteristics. Categorical variables were expressed as counts and percentages, whereas continuous variables were summarized using medians and ranges. A weekly epidemic curve was constructed based on the reported dates of symptom onset and the presumed date of exposure. Because the exact dates of symptom onset were sometimes uncertain, the week was used as the unit of time. All analyses were performed using R version 2024.09.1 (R Foundation for Statistical Computing, Vienna, Austria).

## Results

### Descriptive epidemiology

Approximately 50 individuals attended the festivities each day, of whom 40 were interviewed. A total of 19 individuals subsequently developed symptoms compatible with neuroangiostrongyliasis. Four were hospitalized, 13 presented to the local health center, and 2 were identified retrospectively through participant interviews despite not having sought medical care. Three cases were classified as confirmed, corresponding to the initially hospitalized patients who underwent lumbar puncture and PCR testing. No additional lumbar punctures were performed among the remaining symptomatic individuals; therefore, no cases were classified as probable and 16 were classified as suspected. Among these suspected cases, 7 were classified as high suspicion (3/3 minor criteria), 8 as moderate suspicion, and 1 as low suspicion (Fig. 1). Among the eight moderately suspected cases, six did not present with hypereosinophilia; however, in five of them, eosinophil counts were performed more than 40 days after symptom onset. For the low-suspicion case, neither serology nor blood counts could be performed (Table 1).

**Table 1:** Demographic characteristics, case-finding method, clinical manifestations, laboratory findings, and final case classification among 19 suspected or confirmed cases of neuroangiostrongyliasis identified during the Lifou outbreak investigation

Study ID	Sex	Age	Case source	Symptoms	Onset delay (days)	CSF PCR	CSF eosinophils (cells/mm^3^)	Blood eosinophilia (cells/mm^3^; day)	AC serology	Minor criteria	Case classification
6S	F	48	HOSP	Headache, right arm sensory deficit, diarrhea at onset, infectious colitis with systemic inflammation	10	NA	NA	0 (day 0)	+	2/3	Moderate suspicion
18S	F	59	CONS	Facial nerve palsy, then headache	12	NA	NA	**770** (day 1)	− (× 2)	2/3	Moderate suspicion
1C	M	10	HOSP	Headache, vomiting, meningeal signs, diarrhea at onset	13	Pos	122	**1230** (day 3)	+++	3/3	Confirmed
3C	M	27	HOSP	Headache, neck pain	14	Pos	1790	**760** (day 10)	++	3/3	Confirmed
2C	M	47	HOSP	Headache, vomiting, upper limb pain with sensory deficit, diarrhea at onset	14	Pos	5500	**1440** (day 2)	+++	3/3	Confirmed
8S	F	42	CONS	Headache	18	NA	NA	NA	NA	1/2	Low suspicion
7S	M	14	CONS	Headache, vomiting	18	NA	NA	420 (day 53)	+++	2/3	Moderate suspicion
9S	M	33	CONS	Headache, thigh paresthesia	20	NA	NA	**560** (day 51)	++	3/3	High suspicion
5S	M	15	CONS	Headache, vomiting, migratory limb pain, abdominal pain	20	NA	NA	**1090** (day 8)	+	3/3	High suspicion
4S	F	53	CONS	Headache, vomiting, migratory back and trunk pain	20	NA	NA	**980** (day 8)	+	3/3	High suspicion
11S	M	21	CONS	Headache, vomiting	20	NA	NA	430 (day 240)	++	2/3	Moderate suspicion
10S	M	26	CONS	Headache, vomiting, neck pain	20	NA	NA	400 (day 51)	+	2/3	Moderate suspicion
12S	F	34	CONS	Sciatica-like pain	21	NA	NA	**810** (day 3)	NA	2/2	Moderate suspicion
17S	M	26	RETRO	Headache, vomiting	22	NA	NA	**540** (day 6)	++	3/3	High suspicion
14S	F	19	CONS	Headache	24	NA	NA	**1450** (day 4)	+	3/3	High suspicion
15S	F	60	CONS	Headache, neck pain	26	NA	NA	**630** (day 2)	+++	3/3	High suspicion
16S	F	24	RETRO	Headache, vomiting	29	NA	NA	340 (day 42)	+	2/3	Moderate suspicion
13S	F	8	CONS	Headache	31	NA	NA	190 (day 40)	+	2/3	Moderate suspicion
19S	F	26	CONS	Headache	44	NA	NA	**610** (day 1)	+++	3/3	High suspicion

This table summarizes all suspected and confirmed cases identified during the outbreak investigation. It presents demographic characteristics, case-finding method, diagnostic criteria (including suggestive clinical manifestations and their time of onset), cerebrospinal fluid (CSF) PCR results when available, peripheral eosinophilia together with the interval between symptom onset and blood sampling, bold values indicate peripheral blood eosinophilia (>500 eosinophils/mm³), serological findings for *Angiostrongylus cantonensis*, and the final case classification according to the study case-definition criteria

*ID* case identifier, *CONS* consultation, *HOSP* hospitalization, *RETRO* retrospective interview, *CSF* cerebrospinal fluid, *PCR* polymerase chain reaction, *NA* not available, *AC Angiostrongylus cantonensis* serology: + weakly positive, ++ positive, +++ strongly positive, − negative, −(× 2) negative on two serological tests performed several months apart

The most frequent manifestation was isolated headache, reported in 16 cases. In some patients, headache was associated with vomiting (*n* = 9) or with additional neurological symptoms, including paresthesias or erratic pain (*n* = 4), meningeal signs (*n* = 1), and facial paralysis (*n* = 1).

Two patients presented with atypical, isolated manifestations: one with atypical sciatica and another with transient, isolated upper limb paralysis (Table 1, Fig. S1A). Among the 19 identified cases, three were considered uncertain upon retrospective review.

### Date of exposure and epidemiological curve

All symptomatic individuals had attended the festivities on December 27, which were therefore considered the most likely exposure period (Table S2). The interval between exposure and symptom onset ranged from 2 to 6 weeks, with a median of 3 weeks (IQR: 2–4) (Fig. S1B).

### Investigation of food, water, and environmental exposures

**Fig. 2:**
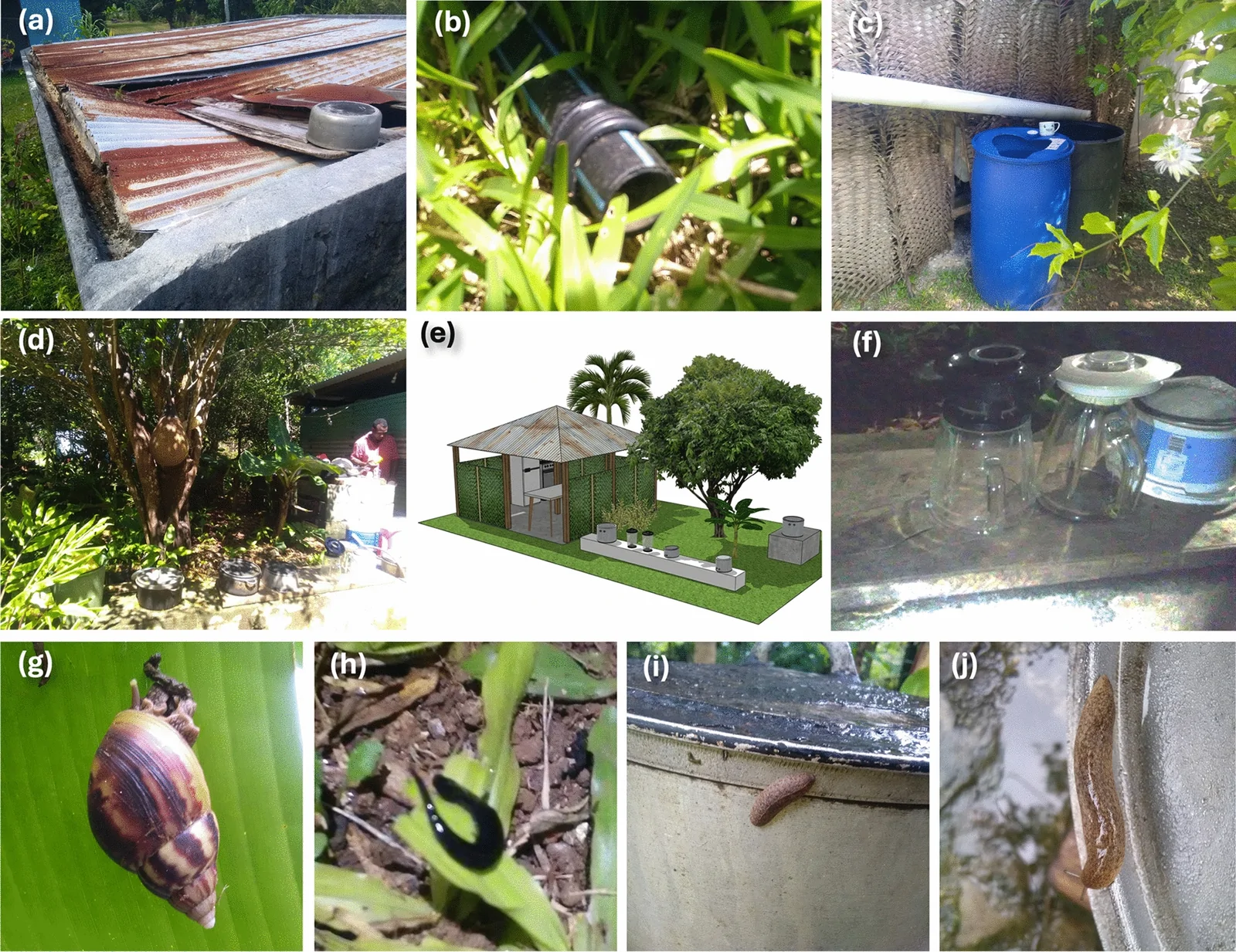
Environmental investigation and potential sources of contamination. **a** Large rainwater storage tank located next to the shelter where the festivities took place. Participants collected water from this tank for drinking at the table and for preparing lemonade. Water was collected from the surface using the pot visible in the photograph; **b** End of a garden hose lying directly on the ground, supplied with treated borehole water. Individuals, particularly those engaged in physical activity during the day, drank directly from it; **c** Rainwater collection barrel located at the home of the person who prepared the juices, from which approximately 7 L of water were taken to dilute the blended fruits; **d** Area adjacent to the traditional Melanesian kitchen where washed dishes were left to dry on a low wall; dishwashing was performed at the washing area visible on the right; **e** Three-dimensional reconstruction illustrating the open kitchen environment within dense vegetation, including the outdoor dishwashing area (left) and the wall used for drying utensils; **f** The two blender bowls (one white, one black) placed on the wall and left to dry after washing; **g**, **h** *Achatina fulica* snails and *Platydemus manokwari* flatworms observed in the vegetation surrounding the kitchen and dishwashing/drying areas; **i**, **j** *Laevicaulis alte* slugs observed on dishes drying on the wall

The preliminary food exposure investigation identified five potential candidates (Table S3): (I) directly consumed garden hose water (Fig. 2b), (II) lemonade diluted with rainwater, (III) raw grated green papaya salad (*Carica papaya*), (IV) locally known *Syzygium malaccense* (“Kanak apple”), and (V) homemade blended fruit juice diluted with rainwater and containing bananas (*Musa* spp.), ripe papaya (*Carica papaya*), passion fruit (*Passiflora edulis*), and lemons (*Citrus limon*).

Further analysis of food consumption histories and participant timelines progressively narrowed the hypotheses to two possible sources: (i) a blended fruit juice diluted with rainwater collected in a large plastic rainwater storage drum (Fig. 2c, consumed by all cases except one), and (ii) a lemonade diluted with rainwater from a large concrete rainwater cistern covered with metal sheets (consumed by all cases, Fig. 2a), both beverages having been prepared using rainwater stored in non-hermetic containers located near the food preparation area.

The environmental investigation revealed rat traces (feces) and observations of mollusks around both water reservoirs. In the analyzed water samples, no larvae or *Angiostrongylus* DNA were detected.

The blended juices were prepared in a traditional open-air kitchen. Clean dishes, including the blender used for juice preparation, were left to dry outside each evening on a small wall near the food preparation area (Fig. 2d, e). Numerous gastropods (*Achatina fulica*, *Laevicaulis alte*, *Platydemus manokwari*) were observed in the vegetation surrounding the kitchen and occasionally directly on drying utensils, particularly *Laevicaulis alte* (Fig. 2g–j).

**Fig. 3:**
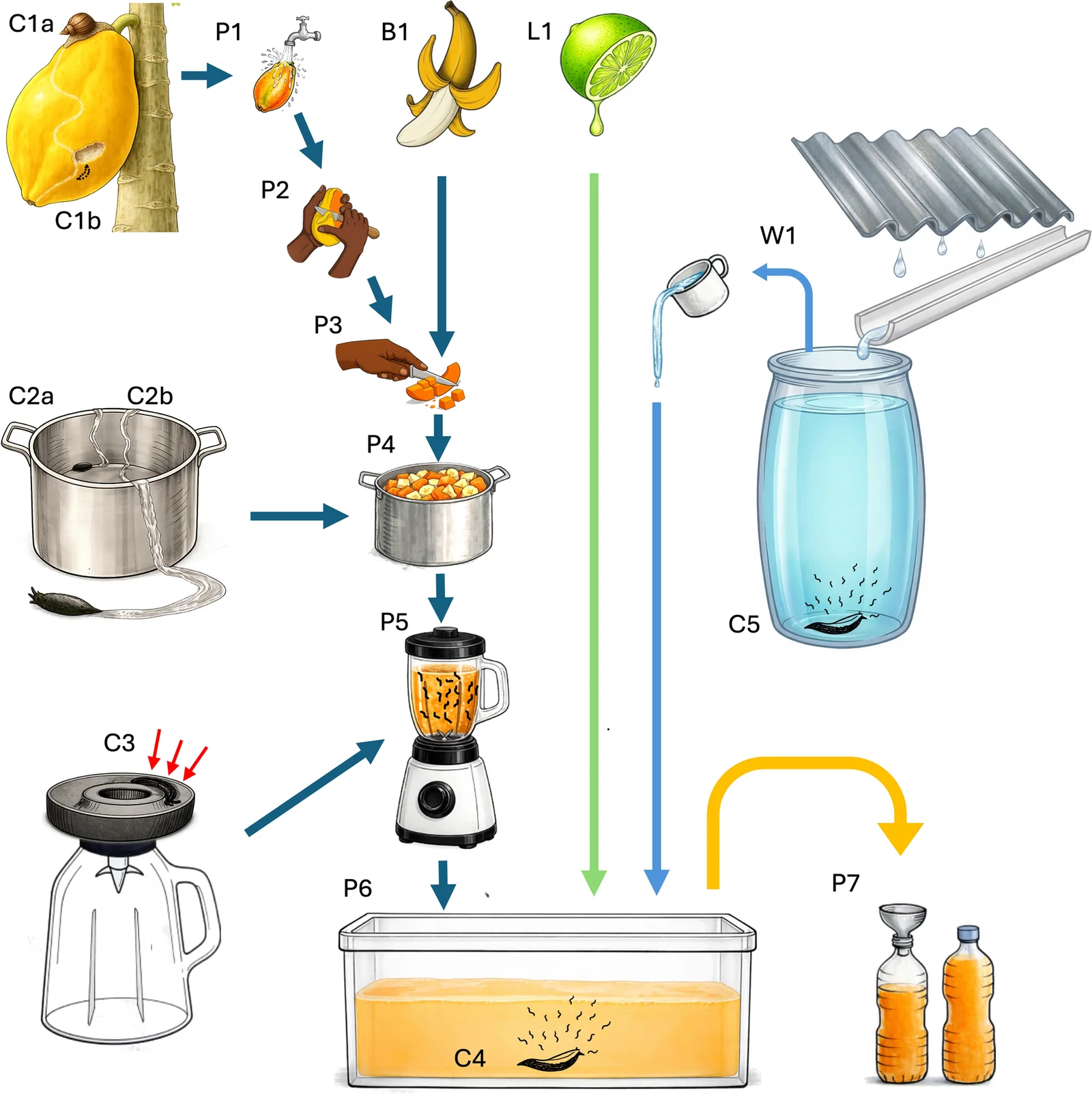
Reconstruction of fruit juice preparation and potential contamination pathways leading to infection. P1–P7, W1, B1, L1 depict the preparation of the fruit juice: papayas were washed, peeled, cut, and blended before being diluted with rainwater (W1) and bottled. Bananas (B1) were processed similarly, and lime juice (L1) was added. Potential contamination routes are indicated: contamination of fruits (C1a–C1b), utensils during drying (C2a–C2b) and blender lid (C3), introduction of a slug into the cooler (C4), or contamination of rainwater (C5), all potentially leading to dissemination of infective L3 larvae

The juice preparation process was further investigated through interviews and reconstruction with the food preparers (Fig. 3).

### Serological findings

Serology for *A. cantonensis* was positive in all confirmed cases (3/3) and in 13 of 14 tested suspected cases; two suspected cases were not tested. All ten exposed but asymptomatic individuals were also seropositive. Among these 27 individuals, 9 (33%) had positive serology for toxocariasis and one for strongyloidiasis.

## Discussion

With 19 cases, this cluster appears to be among the largest reported to date in the literature [16–22], providing a rare opportunity to explore the mechanisms of transmission of a disease in which the route of contamination often remains unclear.

Although the source of infection could not be formally demonstrated, the investigation identified two main hypotheses: contamination of rainwater storage systems and, more likely, contamination of a homemade blended fruit juice prepared during the festivities.

### Waterborne transmission through rainwater used in beverages

Rainwater used to dilute both lemonade and fruit juices could theoretically have been responsible for the contamination. It should be noted that in Lifou, rainwater is frequently preferred to tap water—even though the latter is available in most households—because its chlorinated taste is often perceived as unpleasant.

The hypothesis of waterborne contamination has been raised several times in Hawaii, particularly in connection with rainwater catchment systems [4]. Mollusks are frequently attracted to humid environments and may fall into water tanks, sometimes releasing hundreds of larvae contained in their tissues, which can survive for 21 days in water [4, 23].

However, several observations argue against this hypothesis. When inhabitants collected water from these reservoirs, they generally did so from the surface using a container. The few available studies suggest that larvae are more likely to accumulate at the bottom of the tanks because of sedimentation [4]. Even if the larvae had been evenly distributed, the large dilution volumes involved (approximately 200 and 20,000 L) would likely have resulted in an infective dose too low to infect such a large number of individuals.

Moreover, since larvae can survive for several days, one would expect the contamination period to extend over several days. This does not appear to have been the case. For instance, 2 days after the presumed date of contamination, around thirty individuals from neighboring tribes were invited and also consumed the juices and lemonade, yet none developed symptoms.

Finally, it should be recalled that waterborne transmission, although biologically plausible, has never been formally demonstrated, except in a few isolated cases [4].

### Contamination of blended fruits by mollusks or flatworms

Contamination of the blended fruit juice by mollusks or flatworms prior to dilution appears to be the most likely hypothesis. An association between the consumption of vegetarian smoothies and cases of eosinophilic meningitis has previously been reported in Taiwan [18].

Two potential routes of contamination can be considered: contamination of the fruits themselves or contamination of kitchen containers and utensils. Three mechanisms are theoretically possible: deposition of contaminated mucus [24], release of larvae from a drowned mollusk [4], or the accidental blending of an entire mollusk [18].

The “contaminating slime trail” hypothesis suggests that infective third-stage larvae (L3) originating from an infected mollusk could be released into its mucus and deposited on a vegetable, a glass, or a kitchen utensil [25]. Papayas are known to be visited and sometimes damaged by *Achatina* snails that climb papaya trees to feed on them (Fig. 3), and the investigation showed that mollusks circulated freely around and sometimes on kitchen equipment. However, this mechanism remains debated. Experimental studies suggest that the number of L3 larvae present in the mucus of unstressed mollusks is generally too low to represent a sufficient infective dose, particularly considering that the total volume of juice prepared was approximately 10 L [12, 26]. In addition, the fruits used were intact and were carefully washed and peeled before blending (Fig. 3).

Another hypothesis is the release of larvae by a mollusk that had fallen into the blended juice or the lemonade. This mechanism has been suggested in a cluster reported in the Pacific among individuals consuming kava, a traditional beverage prepared from the roots of the pepper plant [27]. In that case, a snail was found drowned at the bottom of a traditional wooden bowl (*tanoa*) used for kava preparation. In our cluster, however, it seems unlikely that a mollusk could have escaped the attention of the food preparers. The 3 L of blended fruits were poured into a large white-bottomed cooler and then diluted with 7 L of water (Fig. 3). The mixture was immediately transferred into transparent 1.5-L bottles using a funnel and closed with screw caps. Very small specimens might theoretically have gone unnoticed, but the release of larvae from mollusk tissues appears to be uncommon during the first 24 h, which is much longer than the delay between preparation and consumption of the juice [4]. It should also be noted that *Parmarion martensi*, a small slug capable of harboring thousands of larvae, is not present in New Caledonia.

Another possibility is that an entire mollusk was accidentally blended. It is known that *Achatina* snails may climb papaya trees to feed. However, according to the person who prepared the fruits, the papayas were intact and the fruits were peeled and cut before blending (Fig. 3). The presence of a mollusk, usually dark in color, within yellow fruit flesh would have been difficult to overlook.

The final hypothesis, which we consider the most plausible, is that a mollusk or another small organism was introduced directly into the blender. The blender and other kitchen utensils were routinely left outside overnight to dry on a low wall near the kitchen, where numerous gastropods were observed. A mollusk or flatworm could therefore have entered the blender lid during the night and remained unnoticed until the preparation of the juice. If subsequently crushed during blending, this could have led to the release of a large number of larvae.

Among the organisms that might have escaped the attention of the preparers, the flatworm *Platydemus manokwari*, which is very dark in color and could have been poorly visible on the black lid, may be suspected. This flatworm, which was observed near the areas where dishes were drying, is a predator of mollusks and may, like other planarians, harbor infective L3 larvae [23, 24, 28]. This invasive species has been suspected of contributing to several infections in Japan since its introduction [29]. It has been present in New Caledonia since the 1990s. It should also be recalled that endemic planarians were previously considered by Ash to be responsible for infections in New Caledonia [12].

*Platydemus manokwari* frequently lives close to human dwellings and may enter kitchens or be found on vegetation and kitchen utensils while hunting mollusks [28]. It is therefore conceivable that an individual hidden in a fold of the blender lid could have gone unnoticed.

However, the involvement of a flatworm in this scenario is limited by the issue of infective dose. Infection with *A. cantonensis* results from ingestion of infective third-stage larvae (L3), and clinical disease is generally associated with the ingestion of multiple larvae. The minimal number of larvae capable of producing symptoms in humans remains unknown [24, 30]. In rats, it is estimated to be around 30 larvae, but experimental studies in non-permissive hosts such as pigs or calves suggest that several hundreds or even thousands may be required. Extrapolation to humans therefore remains uncertain.

If we assume that the 10 L of juice (3 L obtained by blending and 7 L of added water) were consumed by approximately 40–50 individuals (corresponding to roughly 200–250 ml per person) and that the infective dose is at least around 20 larvae, the crushed mollusk would have needed to contain at least about 1000 larvae. Data on larval burdens for *P. manokwari* and other planarians remain scarce. Available studies reported maximum observed burdens ranging from 81 to 194 larvae per flatworm, although higher burdens cannot be excluded**.** [12, 28]. In contrast, *Laevicaulis alte*, which was abundant at the site, may harbor very high parasite loads, with reports of up to 8600 larvae [12], and could therefore be more likely to produce such a cluster. This order-of-magnitude reasoning suggests that the involvement of a heavily infected gastropod is more plausible than that of a flatworm.

### Case classification and diagnostic uncertainty

In addition to uncertainties related to exposure and diagnostic testing, some limitations regarding case classification should be considered.

Upon retrospective review, three cases were considered uncertain. Case 18S, classified as moderately suspected, presented with facial paralysis and hypereosinophilia but had two negative serological tests and reportedly did not consume the blended fruit juice, the most likely source of exposure. Case 6S presented with transient neurological symptoms without eosinophilia, and an alternative diagnosis (partial seizure in the context of infectious colitis) could not be excluded. Finally, for case 19S, once the presumed date of exposure was established, the delay between exposure and symptom onset (44 days) appeared unusually long.

However, their inclusion is unlikely to have significantly impacted the overall interpretation of the outbreak.

### Serological findings

Serological results suggest that false-positive reactions remain possible in Lifou, particularly for *Toxocara canis*. The 33% seropositivity observed in this study (Fig. S1d) is consistent with toxocariasis serology data from Nouméa Hospital, where a prevalence of approximately 40% has been reported (personal data). However, *Angiostrongylus* antibodies may also cross-react with *Toxocara* antigens.

Finally, positive serological results among exposed but asymptomatic individuals may reflect either a substantial proportion of asymptomatic infections or a high background seroprevalence resulting from previous exposure.

### Study limitations

First, this investigation relied on the reasonable assumption of a single source of contamination; however, multiple sources of exposure cannot be excluded. Second, the delay between the suspected exposure and the field investigation, partly related to the long incubation period, may have affected the accuracy of participants’ recollections and limited the relevance of environmental sampling. Third, information regarding the preparation of the juice was based on the statements of individuals potentially involved in its preparation and may therefore be subject to reporting bias. Fourth, it was not possible to identify and interview all asymptomatic participants who attended the gatherings on December 27 and 28, or to document their potential consumption of the blended fruit juice, thereby hindering the calculation of an attack rate. Fifth, only three lumbar punctures were performed; although this examination represents the only means of confirming the diagnosis or defining a probable case, it is rarely performed when symptoms are limited to isolated persistent headaches without clear meningeal signs. Sixth, the case definition used to identify possible cases may have limitations in endemic settings, as the presence of a single compatible symptom is sufficient for classification as a possible case, and the use of eosinophilia as a minor criterion is also problematic in this setting where several parasitic infections, particularly hookworm infection, are endemic; moreover, eosinophilia was often assessed too late to still be detectable, potentially resulting in underascertainment of this criterion. Seventh, the absence of data on the seroprevalence of *A. cantonensis* in this hotspot of New Caledonia limits the interpretation of serological results, as positive results may reflect previous exposure in an endemic context where seroprevalence may be high (e.g., reaching up to 30% in Hawaii [31]). Finally, the search for *A. cantonensis* larvae in mollusks and rats could not be performed at the time of the investigation; however, environmental sampling conducted 1 year later, including samples collected at the cluster site in December 2024, showed a very high prevalence of *A. cantonensis* in *Achatina fulica* (76%, 32/42) as part of the study “Parasitic meningitis due to *A. cantonensis* in the Pacific: medical awareness and environmental investigation” (preliminary results, unpublished data).

## Conclusion

This study highlights the diagnostic challenges and difficulties in determining the route of contamination for this disease, particularly due to its prolonged incubation period and the absence of a simple confirmatory test for mild clinical forms. Although the source of contamination could not be definitively established, the investigation identified several plausible exposure pathways. Open-air kitchens, commonly used in Melanesian societies, may facilitate food contamination by *A. cantonensis* larvae through the unnoticed presence of small slugs or flatworms on kitchen utensils or food, particularly when food preparation involves shared equipment such as blenders that could potentially amplify exposure. Preventive information regarding these potential risks will be provided to the affected communities. Further seroprevalence studies are planned to better assess the extent of exposure to *A. cantonensis* in the local population.

## Supplementary Information


Supplementary material 1: Table S1: Food and beverage items consumed during the event, categorized by preparation method and origin. Food items were classified according to preparation methodand origin. Highly cooked foods were defined as items subjected to prolonged heating or high-temperature cooking. Uncooked items included foods and beverages consumed without heat treatment, including those prepared with untreated water. Botanical and zoological names are provided where relevant. Beverage items diluted with rainwater or consumed from environmental sources are highlighted due to potential exposure risk.Supplementary material 2: Table S2: Attendance and meal participation of cases during the festive period. Each row represents an individual case. “X” indicates presence at the meal; blank cells indicate absence or no recorded participation.Dates are presented from December 24 to 31, with “L” indicating lunch and “D” indicating dinner.Supplementary material 3: Table S3: Individual consumption of selected food and beverage items among cases. Each row represents a case. “x” indicates reported consumption of the item; blank cells indicate no reported consumption; “?” indicates missing or uncertain information. Food and beverage items were selected based on their potential relevance to exposure. Beverages include those prepared with rainwater or consumed directly from environmental sources.Supplementary material 4: Figure S1A: Clinical presentation of cases. Symptoms are not mutually exclusive. Cervical pain was reported in three patients, including one presenting with meningeal syndrome. Vomiting was reported in nine patients. Figure S1B: Distribution of symptom onset following presumed exposure. Abbreviations: sp = evocative symptoms; sero = positive serology for* Angiostrongylus cantonensis*; Eo = blood eosinophilia.Case classification was defined according to international consensus criteria: confirmed cases required a positive cerebrospinal fluidPCR; probable cases met clinical criteria with CSF eosinophilia and supporting evidence; suspected cases were classified based on the number of minor criteria. The majority of cases occurred between weeks 2 and 4, with a peak at week 3, consistent with the expected incubation period of angiostrongyliasis. Figure S1C: Serological response to *Angiostrongylus cantonensis *among symptomatic and asymptomatic individuals. Serological results among symptomatic and exposed asymptomatic individuals. Black bars represent positive results and white bars represent negative results. Numbers within bars indicate counts. Figure S1D: Serological results for *Toxocara* spp. and *Strongyloides* spp. among all tested individuals. Serological results for *Toxocara* spp. and *Strongyloides* spp. are presented for all individuals tested; brown indicates positive results and white indicates negative results.

## Data Availability

The datasets generated and/or analysed during the current study are not publicly available due to privacy and ethical restrictions but are available from the corresponding author on reasonable request.

## Abbreviations

AC: *Angiostrongylus cantonensis*
CONS: Consultation
CRP: C-reactive protein
CSF: Cerebrospinal fluid
EM: Eosinophilic meningitis
EMR: Electronic medical record
Eo: Eosinophils
HOSP: Hospitalization
ID: Identifier
IQR: Interquartile range
L3: Third-stage larvae
LP: Lumbar puncture
NA: Not available
PCR: Polymerase chain reaction
RETRO: Retrospective interview
Sero: Serology
Swiss TPH: Swiss Tropical and Public Health Institute
