# Supplementary material for: Cluster of 19 cases of eosinophilic meningitis due to Angiostrongylus cantonensis in Lifou, New Caledonia: an outbreak investigation
Source: Infect Dis Poverty. 2026 Sep 1;15:94. doi: 10.1186/s40249-026-01498-7 (PMC13531922; doi:10.1186/s40249-026-01498-7)
Supplement: Supplementary file 1 [file 40249_2026_1498_MOESM1_ESM.docx]

**Additional file 1:**

**Table S1: Food and beverage items consumed during the event, categorized by preparation method and origin**

Food items were classified according to preparation method (uncooked, cooked, highly cooked) and origin (industrial vs local). Highly cooked foods were defined as items subjected to prolonged heating or high-temperature cooking. Uncooked items included foods and beverages consumed without heat treatment, including those prepared with untreated water.

Botanical and zoological names are provided where relevant. Beverage items diluted with rainwater or consumed from environmental sources are highlighted due to potential exposure risk.

| **FOOD INDUSTRY PRODUCTS** | | |
| --- | --- | --- |
| **cooking method** | **item** | **categories** |
| Cooked foods | Rost chiken | Meat and fish |
| Cooked foods | Seawater shrimp | Meat and fish |
| Cooked foods | Sardine soup | Meat and fish |
| Cooked foods | Chips in a bag | Snacks |
| Cooked foods | Potato fries. | Starches |
| Cooked foods | Rice *Oryza sativa* | Starches |
| Uncooked foods | Soda (Coca, Tulem...) | Beverages |
| Highly cooked foods | Boiled cofee | Beverages |
| Highly cooked foods | Beef stew | Meat and fish |
|  |  |  |
| **LOCAL PRODUCTS** | | |
| **cooking method** | **item** | **categories** |
| Uncooked foods | Juice (Passion fruit*, Passiflora edulis*, lemon, banana, papaya *Carica papaya*) diluted with rainwater | Beverages/ Fruits |
| Uncooked foods | Lemonades with rainwater | Beverages/ Fruits |
| Uncooked foods | Iced water (from the Pasko Utramadra tank) | Beverages/ Fruits |
| Uncooked foods | Drinking water from the water supply system, consumed directly from the garden hose laid on the ground (for volleyball players only) | Beverages/ Fruits |
| Uncooked foods | Green papaya salad | Vegetables |
| Uncooked foods | **"Kanak apples"** directly or in salad form, *Syzygium malaccense* | Fruits |
| Cooked foods | Cakes | Desserts |
| Cooked foods | lobster *Homarus gammarus* | Meat and fish |
| Cooked foods | Grilled fish | Meat and fish |
| Cooked foods | Roast pork | Meat and fish |
| Highly cooked foods | Banana gratin | Fruits |
| Highly cooked foods | Spectacled flying fox *(Pteropus conspicillatus)* | Meat and fish |
| Highly cooked foods | **"Bougna",** ingredients are wrapped in banana leaves and stewed in a kanak oven | Starches |
| Highly cooked foods | Taro, *Colocasia esculenta* | Starches |
| Highly cooked foods | **"Wed"**, boiled ferns *asplenium nidus* | Vegetables |
| Highly cooked foods | **"Pahtr"**, fried ferns, *asplenium nidus* | Vegetables |
