# Supplementary material for: Cluster of 19 cases of eosinophilic meningitis due to Angiostrongylus cantonensis in Lifou, New Caledonia: an outbreak investigation
Source: Infect Dis Poverty. 2026 Sep 1;15:94. doi: 10.1186/s40249-026-01498-7 (PMC13531922; doi:10.1186/s40249-026-01498-7)
Supplement: Supplementary file 2 [file 40249_2026_1498_MOESM2_ESM.docx]

**Additional file 2:**

**Table S2. Attendance and meal participation of cases during the festive period (December 24–31).** Each row represents an individual case. Confirmed cases are highlighted in yellow. Presence (✓) at the banquet site and participation in meals are shown for each day between December 24 and 31. “Day” denotes attendance at the banquet site without documented meal consumption. Individuals may nevertheless have been exposed through beverages, including rainwater, water consumed directly from a garden hose, or blended fruit juices. “Lunch” denotes participation in the midday meal, and “Dinner” denotes participation in the evening meal. The red and orange columns indicate the two exposure windows considered the most likely periods of contamination based on the epidemiological investigation.
