# Supplementary material for: Cluster of 19 cases of eosinophilic meningitis due to Angiostrongylus cantonensis in Lifou, New Caledonia: an outbreak investigation
Source: Infect Dis Poverty. 2026 Sep 1;15:94. doi: 10.1186/s40249-026-01498-7 (PMC13531922; doi:10.1186/s40249-026-01498-7)
Supplement: Supplementary file 3 [file 40249_2026_1498_MOESM3_ESM.docx]

**Additional file 3:**

**Table S3: Individual consumption of selected food and beverage items among cases**

Each row represents a case. “x” indicates reported consumption of the item; blank cells indicate no reported consumption; “?” indicates missing or uncertain information.

Food and beverage items were selected based on their potential relevance to exposure. Beverages include those prepared with rainwater or consumed directly from environmental sources (cistern or

| study ID | **Food items** | | |  | **Beverage** | | | |
| --- | --- | --- | --- | --- | --- | --- | --- | --- |
|  | Pacific rose apple | rose apple salad | green papaya salad |  | blended fruit juice | homemade lemonade | cistern rainwater | garden hose |
| 13S | x | x |  |  | x | x | x | x |
| 1C | x | x | x |  | x | x | x | x |
| 7S | x | x | x |  | x |  | x | x |
| 5S | x | x |  |  | x | x | x | x |
| 14S | x | x |  |  | x | x | x |  |
| 11S | x | x | x |  | x | x | x | x |
| 16S | x | x | x |  | x | x | x |  |
| 17S | x | x |  |  | x | x | x | x |
| 19S | x | x | x |  | x | x | x |  |
| 10S | x | x | x |  | x | x | x | x |
| 3C | x |  |  |  | x | x | x | x |
| 9S |  |  |  |  | x | x | x | x |
| 12S | x | x | x |  | x | x | x |  |
| 2C | x | x | x |  | x | x | x |  |
| 6S | x | x | x |  | x | x | x |  |
| 4S | x | x | x |  | x | x | x |  |
| 18S | x |  |  |  |  | x | x |  |
| 15S | x | x | x |  | x | x | x |  |
| 8S | ? | ? | ? |  | ? | ? | ? | ? |
