# Supplementary material for: Cluster of 19 cases of eosinophilic meningitis due to Angiostrongylus cantonensis in Lifou, New Caledonia: an outbreak investigation
Source: Infect Dis Poverty. 2026 Sep 1;15:94. doi: 10.1186/s40249-026-01498-7 (PMC13531922; doi:10.1186/s40249-026-01498-7)
Supplement: Supplementary file 4 [file 40249_2026_1498_MOESM4_ESM.docx]

**Additional file 4:**

**Figure S2A: Clinical presentation of cases (n=19)**

Symptoms are not mutually exclusive. Cervical pain was reported in three patients, including one presenting with meningeal syndrome. Vomiting was reported in nine patients.

**Figure S2B: Distribution of symptom onset following presumed exposure**

Abbreviations: sp = evocative symptoms; sero = positive serology for Angiostrongylus cantonensis; Eo = blood eosinophilia (>500 cells/mm³).Case classification was defined according to international consensus criteria: confirmed cases required a positive cerebrospinal fluid (CSF) PCR; probable cases met clinical criteria with CSF eosinophilia and supporting evidence; suspected cases were classified based on the number of minor criteria (exposure, eosinophilia, serology). The majority of cases occurred between weeks 2 and 4, with a peak at week 3, consistent with the expected incubation period of angiostrongyliasis.

**Figure S2C: Serological response to Angiostrongylus cantonensis among symptomatic and asymptomatic individuals**. Serological results among symptomatic and exposed asymptomatic individuals. Black bars represent positive results and white bars represent negative results. Numbers within bars indicate counts.

**Figure S2D: Serological results for Toxocara spp. and Strongyloides spp. among all tested individuals (potential cross-reactivity).**

Serological results for Toxocara spp. and Strongyloides spp. are presented for all individuals tested; brown indicates positive results and white indicates negative results.
